# Evaluation of Specific Contour Propagation Tool Accuracy for Lung Tumor Lesions

**DOI:** 10.7759/cureus.81913

**Published:** 2025-04-08

**Authors:** Hideaki Matsukawa, Eiji Shiba, Ryohei Kuroki, Yoshio Iwai, Takayuki Ohguri, Takatoshi Aoki

**Affiliations:** 1 Radiology, University of Occupational and Environmental Health Hospital, Kitakyushu, JPN; 2 Research Physics, Elekta KK, Tokyo, JPN; 3 Therapeutic Radiology, University of Occupational and Environmental Health Hospital, Kitakyushu, JPN

**Keywords:** adaptive radiation therapy, automated contouring, cone-beam computed tomography, deformable image registration (dir), lung cancer, propagation, treatment planning system

## Abstract

Introduction: Adapt Anatomy is an automatic propagation tool installed in the Monaco^®^ treatment planning system v. 5.5.1 (Elekta AB, Stockholm, Sweden). Few studies have reported the usefulness of Adapt Anatomy for adaptive radiation therapy (ART) in clinical cases. This study aimed to investigate the accuracy of Adapt Anatomy for lung tumor lesions.

Methods: Ten patients with lung cancer were treated at our hospital, and treatment plans were formulated again during the radiotherapy course. Each patient’s gross tumor volume (GTV), lung, cord, heart, and esophagus were contoured manually by radiation oncologists and automatically by Adapt Anatomy on replanning computed tomography (rCT) images. The structures were also contoured on cone-beam computed tomography (CBCT) images obtained on the same days as the rCT images, using Adapt Anatomy. The structural volumes contoured using Adapt Anatomy were compared with those contoured by the radiation oncologists. The dice similarity coefficient (DSC) and overlap index (OI) were calculated for each structure. Additionally, the DSC and OI calculated for the GTV on the CBCT images were compared with the values calculated from the rCT images.

Results: The automatic contour volumes for all of the structures did not differ significantly from the manual contour volumes, except for heart and esophagus volumes. The DSC and OI values for all of the structures were >0.8, except for the esophagus. The DSC and OI values for the esophagus were 0.73±0.070 and 0.68±0.10, respectively. The DSC and OI values calculated for the CBCT images did not differ significantly from those calculated for the rCT ones (p=0.208, 0.401, respectively).

Conclusion: Adapt Anatomy showed sufficient accuracy compared to the other software solutions, and we concluded that there is a possibility that Adapt Anatomy may be useful for performing ART on CBCT images. However, careful reviews by radiation oncologists are warranted in clinical cases because its accuracy varies significantly in certain structures such as the esophagus.

## Introduction

Image-guided radiation therapy (IGRT) has been widely used to assess and reduce the inter-fractional setup variations and internal organ motion and deformation for high-precision radiotherapy [1-3]. In current standard clinical IGRT practice, cone-beam computed tomography (CBCT) scans are taken to correct the inter-fractional target displacement, and show potential in terms of personalizing radiation therapy delivery according to the anatomical changes identified in each patient [4-6]. This treatment strategy is known as adaptive radiation therapy (ART), and is used to change treatment plans by assessing the dose distribution calculated via computed tomography (CT) or image guidance systems during treatment courses, as necessary [7-9]. In other words, the aim of ART is to adapt treatment plans in order to improve tumor coverage and organ sparing according to anatomical changes such as tumor shrinkage. Repeat imaging and replanning, even with a single mid-treatment scan, significantly improved tumor coverage and organ sparing for patients who experienced clinically apparent changes in anatomy [10,11]. Tumor volume shrinkage during treatment for lung cancer is a well-known phenomenon that makes approaches such as ART highly attractive [12]. Treatment replanning is implemented for lung tumor lesions that shrink in volume following radiotherapy, at our institutes.

Monaco® treatment planning system v. 5.5.1 (Elekta AB, Stockholm, Sweden) is equipped with an automatic propagation tool “Adapt Anatomy”, which uses deformable image registration (DIR) to support ART. Propagation is done by contouring structures on the target image based on another image of the same patient as a type of automatic contouring. Segmentation is also performed as another automatic contouring method based on imaging data from different patients. The increasing demand for volume delineation in conformal and adaptive radiotherapy has led to a growing selection of automatic contouring software, as was detailed in a report by La Macchia et al., where software solutions were evaluated quantitatively both in terms of speed and reliability [13]. ART requires multiple physician-drawn volumes, thereby increasing the workload on already limited physicians. If the robust accuracy of Adapt Anatomy can be demonstrated, it could potentially alleviate the burden. In addition, replanning decisions can be quantitatively evaluated according to dose volume histogram (DVH) and dose distribution by using Adapt Anatomy for CBCT images and dose calculation. CBCT images have lower contrast than CT ones, and image artifacts and noise degrade image quality. These features can affect the accuracy; therefore, it is important to investigate the propagation accuracy for CBCT images. However, few studies have reported the usefulness of Adapt Anatomy for ART in clinical cases [14], and no study has reported it for CBCT images, to our knowledge. In this study, we evaluated how well the Adapt Anatomy creates structures-including gross tumor volume (GTV) and organs at risk (OAR) on CT images to CT or CBCT images. Therefore, we aimed to investigate the accuracy of Adapt Anatomy for lung tumor lesions. However, it should be noted that this study has limited clinical applicability because Adapt Anatomy is a unique function of Monaco, not other treatment planning systems.

## Materials and methods

This retrospective study was approved by the Ethics Committee of Medical Research of the University of Occupational and Environmental Health (approval number: R3-049).

**Figure 1:**
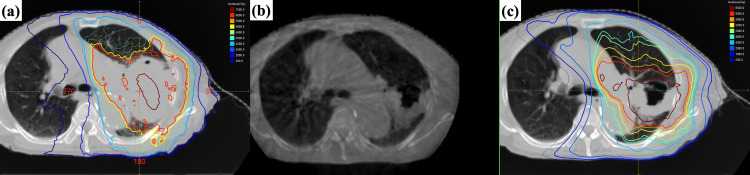
An example of adaptive radiation therapy performed for lung cancer (a) Dose distribution on the initial planning CT, (b) Cone-beam CT image at 44 Gy, and (c) Dose distribution on the replanning CT

Between August of 2019 and February of 2022, 10 consecutive patients with lung cancer were treated at the University of Occupational and Environmental Health Hospital, Kitakyushu, Fukuoka, Japan, and their treatment plans were formulated again during the radiotherapy course. Table 1 shows the patient data and their tumor characteristics. The median patient age was 72 years (range, 15-85 years). One of the 10 patients had pulmonary metastasis from Ewing sarcoma, and the other nine had primary lung cancer. Of these nine, five had TNM stage IIIA, one had IIIC, three had IVA, three had tumor-type adenocarcinoma, five had squamous cell carcinoma, and one had small-cell lung cancer (SCLC). Definitive radiotherapy was administered to six patients with non-small cell lung cancer (NSCLC) at a total dose of 60 Gy in 30 fractions. One patient with an NSCLC superior sulcus tumor received 66 Gy in 33 fractions. Additionally, one patient with SCLC received 45 Gy in 30 fractions, delivered as accelerated hyperfractionated radiotherapy. Patients with synchronous oligometastatic NSCLC received 56 Gy in 28 fractions to the primary tumor and regional lymph nodes, while patients with gross pulmonary oligometastases of Ewing's sarcoma received 50.4 Gy in 28 fractions. All treatments followed standard fractionation regimens used in clinical practice. For all of the cases, the initial planning computed tomography (pCT) images and the replanning CT (rCT) images were obtained using a CT Asteion/Multi TSX-021A instrument (Toshiba Medical Systems, Tokyo, Japan), and ART was implemented during the radiotherapy course. Figure 1 shows an example of ART used for lung cancer treatment. 

**Table 1:** Patient and tumor characteristics F: female; M: male; A: adenocarcinoma; S: squamous cell carcinoma; SCLC: small cell lung cancer

Case	Age	Sex	Stage	Histology	Prescription dose (cGy)	Fractions
1	59	F	IVA	A	6000	30
2	85	M	IIIA	S	6000	30
3	28	F	IIIA	A	6600	33
4	73	F	IIIC	S	6000	30
5	72	M	IIIA	A	5600	28
6	72	F	IIIA	S	6000	30
7	76	M	IIIA	S	6000	30
8	60	M	IVA	S	6000	30
9	77	M	IVA	SCLC	4500	30
10	15	F	Pulmonary metastasis from Ewing sarcoma		5040	28

GTV and OAR, including the lungs, spinal cord, heart, and esophagus, were contoured by each radiation oncologist in charge on the patients’ pCT and rCT images using Monaco according to Japanese Society for Radiation Oncology Guidelines for Radiotherapy Treatment Planning, and these structures were checked and corrected by a senior radiotherapy specialist in clinical cases. However, the heart and esophagus were only contoured in eight of the patients. In addition, CBCT images were obtained using X-ray volume imaging (XVI) performed via Versa HD (Elekta AB, Stockholm, Sweden) for image guidance on the same day that the rCT images were obtained in eight of the patients. The CBCT reconstruction parameters of XVI are shown in Table 2 with reference to XVI instructions for use. The Wiener was used as the reconstruction filter, and the interpolation parameter was “Partial2”. For this parameter, XVI first interpolates the projection image by a multiple of two and then does a near-neighbor interpolation along the back-projection. This gives an optimization of the reconstructed image quality, and is the default setting. The reconstruction data type was set to “float”, in which XVI did a slow reconstruction that was of the highest quality. The pre-filter was not set, so the resolution was the highest, and the contrast was the lowest. The contrast adjustment was performed using “Lung” preset on the “Auto Window and Level presets” in XVI at the time of IGRT. The pCT, rCT, and CBCT images obtained during radiation therapy were used for the retrospective analysis conducted in this study.

**Table 2:** The cone-beam CT reconstruction parameters of XVI ReconstrutionVoxelSize=The length, height, and width (in millimeters) of the cubic voxels that make the reconstructed 3D image volumes, in millimeters; ReconstrutionDimensionX, Y, Z=The size, in voxels, of the reconstructed 3D volume in the X, Y, and Z directions; ReconstrutionFilter= The filter used in the filter back-projection procedure of 3D volume reconstruction; ScatterCorrectionParameter1= The scattered radiation to primary radiation ratio; ReconstructionDataType= The accuracy of the data types that XVI uses to do a 3D reconstruction; PreFilter= The filter that XVI applies to the projection images, before it continues to reconstruct the 3D volume; ProjectionDownSizeFactor=The parameter of the speed of the 3D reconstruction and the image quality

Parameter	Value
ReconstrutionVoxelSize	1.0
ReconstrutionDimensionX	270
ReconstrutionDimensionY	264
ReconstrutionDimensionZ	270
ReconstrutionFilter	Wiener
Interpolation	Partial2
ScatterCorrection	Uniform
ScatterCorrectionParameter1	0.24
ReconstructionDataType	float
PreFilter	None
ProjectionDownSizeFactor	2

Adapt Anatomy is an automatic propagation tool that makes use of the DIR that is installed in the Monaco Ver 5.5.1 treatment planning system. It can automatically create structures on rCT or CBCT images based on the structure in the initial pCT image. In this study, the structures were contoured on the rCT image using Adapt Anatomy without manual correction, with reference to the structures contoured on the pCT image. The propagation mode can be selected as deformable or rigid on the “Adapt Setup” tab, and the deformable mode was chosen in all structures. The structures included the GTV, lung (tumor side), lung (normal tissue side), spinal cord, heart, and esophagus. The heart and esophagus were contoured for only eight of the patients, similarly to how the initial treatment plans were constructed based on the pCT images. Additionally, the structures contoured by Adapt Anatomy were blinded when the radiation oncologist contoured structures on rCT images. In order to use Adapt Anatomy, it is necessary to adjust the positions of the pCT and rCT images. These images were overlaid on Monaco using the fusion function, with fusion type set to “Translation and X/Y/Z Rotations”. These images were then combined using automatic rigid fusion and manually corrected in a similar way to the image-guided registration on a CBCT image before irradiation. Specifically, body axes were aligned with bone structures, and the positional shifts were corrected so that the tumor did not exceed the planning target volume (PTV). Furthermore, the structures were automatically contoured on the CBCT images obtained on the same day as the rCT images for eight of the cases without manual correction, based on the pCT images, using Adapt Anatomy. However, only the GTV structures were investigated because they were included in the field of view of the CBCT images. In order to use Adapt Anatomy, it is also necessary to adjust the positions of the pCT and CBCT images. For this phase, the reconstruction slice thickness was set to 2 mm as the pCT image, and the export type was set to “option 3-export reconstructed volume in reference volume coordinated correction Applied” when the CBCT image obtained at the irradiation position by the XVI was exported to Monaco. Thus, the CBCT image was imported into Monaco at a position that had already been corrected.

The structure volumes contoured by Adapt Anatomy and the radiation oncologists were defined as Va and Vm respectively, and the variation rate ΔV (%) [13], dice similarity coefficient (DSC), and overlap index (OI) were calculated for each structure using these parameters, in order to evaluate the contour accuracy of Adapt Anatomy. The DSC is an index that evaluates the degree of contour matching [13,15], and varies between 0-1. The value is identical to 1 if automatic and manual volumes were equal with a complete intersection. Whereas DSC describes volume similarity, OI reflects the inclusion of Vm within Va [16]. The formulas for deriving ΔV, DSC, and OI are defined as:

$\Delta V(\%)=\frac{V_{a}-V_{m}}{V_{m}}\times 100$

$\text{DSC}=\frac{2\left( V_{m} \cap V_{a}\right)}{V_{m}+V_{a}}$

$\text{OI}=\frac{V_{m}\cap V_{a}}{V_{m}}$

The Vm and Va volumes were statistically compared for each structure in this analysis. In addition, the DSC and OI values calculated for GTV on the CBCT images were compared with the values calculated on the rCT images. IBM SPSS Statistics for Windows, Version 27.0 (Released 2020; IBM Corp., Armonk, NY, USA) was used for all statistical analyses, and statistical comparisons were performed using the Wilcoxon signed rank-sum test. Statistical significance was set at p<0.05. 

## Results

The volume parameters (Vm, Va, and ΔV), the contour agreement indexes (DSC and OI) for each structure, and the statistical analysis results are shown in Table 3. The automatic contour volumes (Va) for all of the structures did not differ significantly from the manual contour volumes (Vm), except for the heart and esophagus. Moreover, the mean volume variation rates (ΔVs) of the heart and esophagus were -8.89±7.14% (p=0.025) and -14.03±12.79% (p=0.017), respectively. This result indicates that the automatic contour volumes obtained using Adapt Anatomy tended to be smaller than the manual contour volumes for the heart and esophagus. The mean DSC for the structures (i.e., GTV, lung (tumor side), lung (normal tissue side), spinal cord, heart, and esophagus) were 0.85±0.047, 0.94±0.039, 0.97±0.013, 0.81±0.053, 0.90±0.050, and 0.73±0.070, respectively. Similarly, the mean OI values were 0.87±0.059, 0.94±0.052, 0.97±0.010, 0.81±0.13, 0.86±0.073, and 0.68±0.10, respectively. The DSC values for all of the structures were > 0.8, except for the esophagus, and those for the lungs were >0.9. The DSC esophagus values were 0.73±0.070, which was lower than for the other structures. The OI values were also >0.8, except for the esophagus, and those for the esophagus were lower than for the other structures.

**Table 3:** Statistical comparison between the automatic and manual structure volumes GTV=GTV contoured on the rCT image; GTV (CBCT)=GTV contoured on the CBCT image; p value=p value calculated through a statistical comparison between Vm and Va; p value (rCT-CBCT)=p value calculated through a statistical comparison between DSC and OI for the GTV contoured on the rCT image and for the GTV contoured on the CBCT image GTV: gross tumor volume; rCT: replanning computed tomography; CBCT: cone-beam computed tomography; Vm: structure volume contoured by radiation oncologist; Va: structure volume contoured by Adapt Anatomy; DSC: dice similarity coefficient; ΔV: variation rate

	Vm (cm^3^), mean±SD	Va (cm^3^), mean±SD	ΔV (%), mean±SD	p value	OI, mean±SD	DSC, mean±SD
Lung (tumor side)	1160.42±607.93	1179.52±628.75	0.90±5.95	0.721	0.94±0.052	0.94±0.039
Lung (normal tissue side)	1734.29±597.83	1746.32±594.67	1.27±3.33	0.575	0.97±0.010	0.97±0.013
Spinal cord	31.30±7.38	30.67±9.45	-1.01±26.25	0.799	0.81±0.13	0.81±0.053
Heart	675.59±151.93	619.55±158.41	-8.89±7.14	0.025	0.86±0.073	0.90±0.050
Esophagus	32.22±8.85	26.90±5.25	-14.03±12.79	0.017	0.68±0.10	0.73±0.070
GTV	300.93±243.14	312.12±261.63	3.60±9.13	0.241	0.87±0.059	0.85±0.047
GTV (CBCT)	313.95±260.15	331.33±288.73	3.71±9.48	0.263	0.85±0.073	0.83±0.053
p value (rCT-CBCT)					0.401	0.208

**Figure 2:**
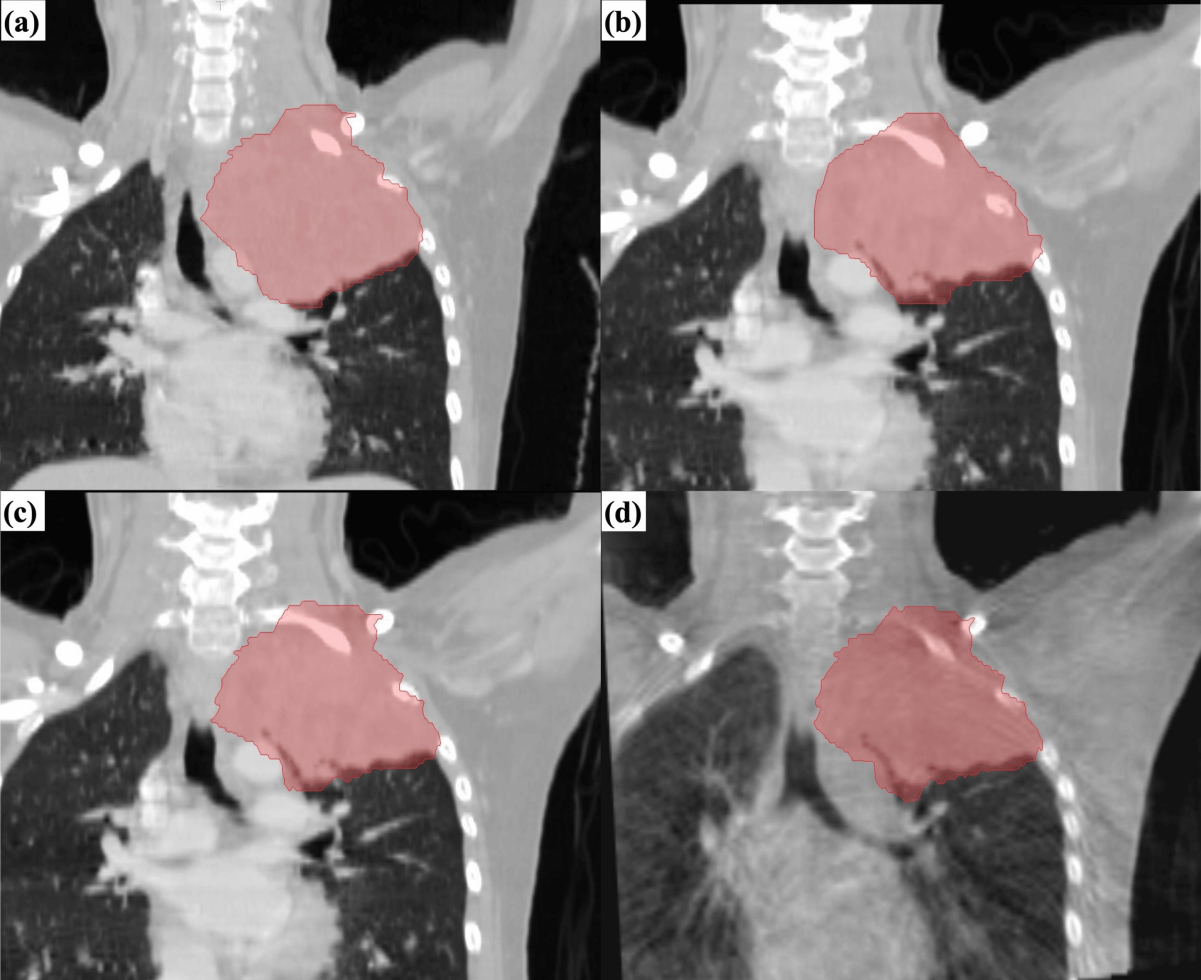
An example of the gross tumor volume structures contoured by Adapt Anatomy for a patient with lung cancer (a) Planning computed tomography contoured by radiation oncologists; (b) Replanning CT contoured by radiation oncologists; (c) Replanning CT contoured automatically by Adapt Anatomy; (d) Cone-beam computed tomography contoured automatically by Adapt Anatomy

For GTV, the DSC values of the rCT and CBCT images were 0.85±0.047 and 0.83±0.053, respectively, and the OI values were 0.87±0.059 and 0.85±0.073, respectively. The DSC and OI values of the CBCT images did not differ significantly from those of the rCT images (p=0.208 and 0.401, respectively). Figure 2 shows an example of the GTV structures contoured using Adapt Anatomy for a patient with lung cancer. Although the GTV edges on rCT and CBCT contoured automatically by Adapt Anatomy are unstable (Figure 2c, 2d), those are approximately the same shape as that contoured by radiation oncologists (Figure 2b).

## Discussion

In this study, we investigated the propagation accuracy of the Adapt Anatomy feature installed on the Monaco treatment planning system for lung cancer. The Va did not differ significantly from Vm, except for the heart and esophagus. For the heart and esophagus, the Va values tended to be lower than the Vm ones. In addition, the DSC values for all of the structures were >0.8, except for the esophagus, and those for the lungs were >0.9. The Va and Vm values did not differ significantly, and high DSC and OI values were observed for the GTV, lungs, and spinal cord. La Macchia et al. evaluated three commercial software solutions for automatic segmentation for adaptive therapy in head-and-neck, prostate, and pleural cancers [13]. They reported that the DSC value was ≥0.8 for the target and ≥0.9 for the lung. McDonald et al. evaluated the quality of contours propagated via the Adapt Anatomy for head and neck cancer, in terms of magnetic resonance (MR) images [14]. They deformed OAR contours (bilateral parotid glands, bilateral submandibular glands, spinal cord, brain stem, and mandible) from CT or MR to MR images. They reported that the median DSC value fell below the threshold value of 0.8 for CT-to-MR in all cases; however, the median DSC met this threshold for MR-to-MR in most cases. 

Considering these reports and our results, Adapt Anatomy also demonstrated sufficient accuracy compared to the other software solutions and devices reported previously [13,14]. For the heart, although the volumes differed significantly, high DSC and OI values (0.90 and 0.86, respectively) were observed. Thus, we concluded that Adapt Anatomy showed adequate accuracy. The DSC tended to be higher when the volume was large; however, high DSC values were also returned for the GTV and spinal cord, despite these having smaller volumes than the other structures. Brock et al. reported the tolerance of DSC (~0.80-0.90) values for the structures [17], and those we measured in our study were within the tolerance limits set, except for the esophagus. The volumes differed significantly between the automatic and manual contours, and the DSC and OI showed the lowest values in the esophagus. The likely reason why the contouring accuracy for the esophagus was inferior to other structures is the absence of consistent intensity contrast between the esophagus and surrounding tissues in thoracic CT scans. In addition, the esophagus has a certain mobility, which yields a very inhomogeneous appearance and a complex shape [18]. Several studies have reported that the esophageal intra-fractional motion caused by respiration and cardiac motion is between 5 and 10 mm [19,20]. We considered that the automatic contour accuracy for the esophagus was lower than for the other structures because the contrast between the esophagus and the surrounding organs is low, and its shape is irregular. Furthermore, the DSC and OI readings for GTV showed high values of ≥0.8, and there was no significant difference between rCT and CBCT in terms of a statistically comparative analysis. Therefore, we concluded that the Adapt Anatomy system achieved similar contouring accuracy on CBCT images as it did on CT ones. However, since the esophagus has a lower DSC and a higher ΔV compared to the other structures, we considered that radiation oncologists should double-check Adapt Anatomy results in clinical cases and correct the contoured structures if necessary. In this study, the threshold value was set at DSC 0.8 in accordance with previous reports [13,14,17]. However, Voet et al. found underdosages in the PTV of up to 11 Gy, even for DSC coefficients of 0.8 for head and neck cancer cases [21]. Although we did not evaluate the dosimetric impact of contour mismatch, we consider that the automated propagation protocol should also be evaluated from this perspective, which is needed for our future research.

Although ART is useful in cases where the tumor shape changes, adaptive planning is required for multiple physician-drawn volumes; thus, the burden on the physician increases. However, this can be reduced by automatically contouring the structures using the Adapt Anatomy propagation tool. Although we could not compare the contouring time between manual and the Adapt Anatomy due to the retrospective nature of this study, several studies have reported that the contouring time was reduced by ~10-30 min using automatic contouring software [22,23]. The automated propagation tool “Adapt Anatomy” is therefore expected to save physicians significant sums of replanning time and burden.

**Figure 3:**
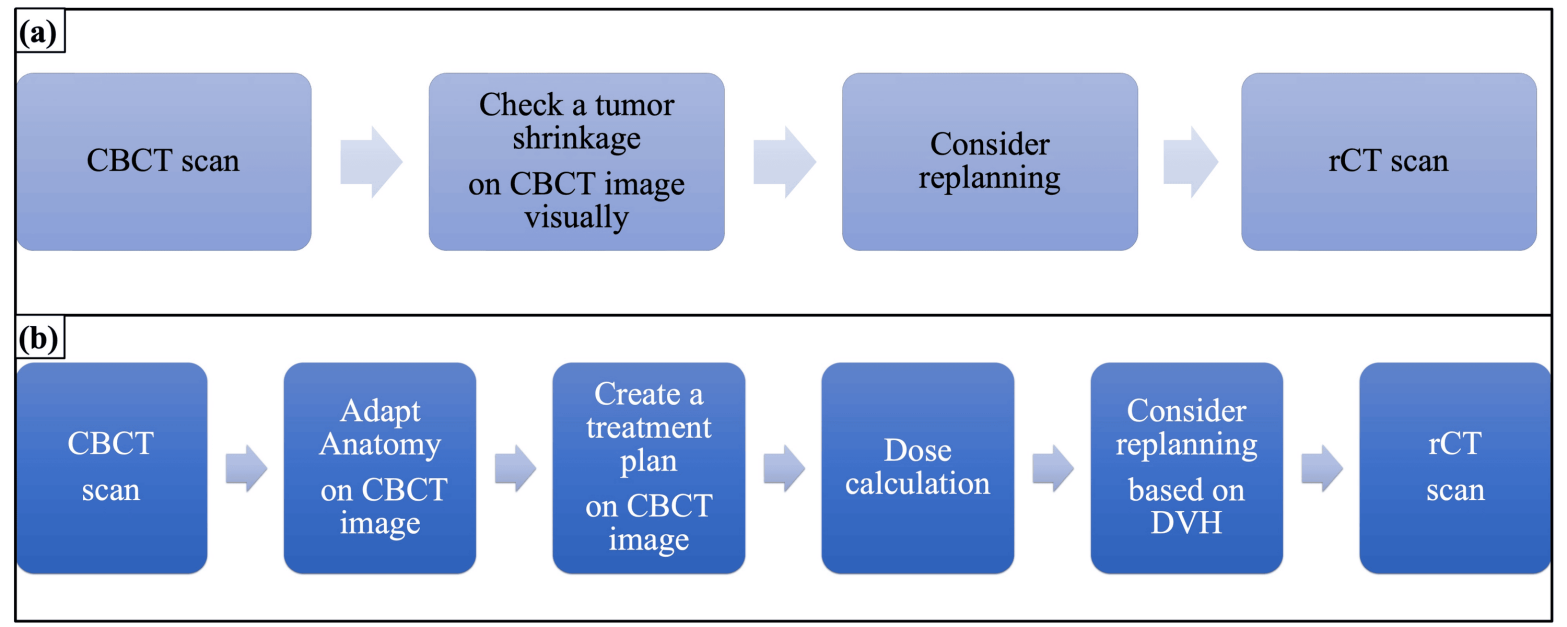
Adaptive radiation therapy workflow using CBCT images acquired at our institute (a) Previous method; (b) Adapt Anatomy method rCT: replanning computed tomography; DVH: dose volume histogram; CBCT: cone-beam computed tomography

Although we concluded that Adapt Anatomy has the possibility to be useful for performing ART on CBCT images, electron density information is essential for calculating the dose distribution on CBCT images in clinical cases. However, CBCT images have a lower contrast than CT ones, as well as reduced image quality caused by imaging artifacts and noise. The pixel values of CBCT images vary significantly because of the scattered photons from the patients [24]. These influence the relationship between CT pixel numbers (Hounsfield Unit) and the electron density. Dose calculation errors are, therefore, introduced when dose calculations are performed on CBCT images. Previous studies have reported that dose differences varied between 0-2.3% when certain methods were used on CBCT images [25,26]. Those studies mentioned that deformable registration represented an accurate method for performing dose calculations on CBCT. In particular, Giacometti et al. indicated that median dose differences fell within 1.2% when density overrides were performed on CBCT images using electron density maps on pCT images [26]. There is an electron density override tool that can replace the density on a CBCT image with a mean electron density on the pCT image in Monaco. Thus, dose distributions on CBCT images can be calculated more accurately by using mean electron densities on the reference CT image for each structure. Figure 3 shows the ART workflow using the CBCT images obtained at our institute. Previously, when deciding whether to perform ART, the only viable method was to visually and qualitatively check the degree of tumor shrinkage on CBCT images. However, we can evaluate the decision to replan quantitatively according to a dose volume histogram and dose distribution by using Adapt Anatomy on the CBCT image and dose calculation. Future research is needed to investigate the effect on dose calculation for CBCT images in our institute, specifically in the context of implementing the aforementioned ART workflow in Monaco.

Limitations of the study and future recommendations

Our study has some potential limitations. First, the sample size was limited because we only focused on cases whose treatment plans were formulated again during the radiotherapy course. Therefore, we used non-parametric tests for statistical analyses. There was no significant difference between DSC and OI on rCT images and those on CBCT images. However, it is possible that the study was underpowered due to an insufficient number of cases. Additionally, the structures contoured by the radiation oncologist were used as the reference in clinical cases that underwent replanning during the treatment course. It is probable that inter-observer variability affects the results; however, the inter-observer variability could not be considered because the structures used in this retrospective study had already been contoured by oncologists and checked and corrected by a senior radiotherapy specialist in the clinical cases. Therefore, further studies involving more cases and multiple institutions are needed. In addition, manual correction is required, using a fusion tool in Monaco, to perform Adapt Anatomy from pCT images to rCT ones. Besides, CBCT images corrected manually in clinical cases were used to perform Adapt Anatomy from pCT images to CBCT ones. Setup variations and subjectivity were included when we carried out a fusion tool in Monaco, owing to manual corrections in the registration of both the rCT and CBCT images. Thus, we indicated the results included these errors because it was impossible to scan rCT and CBCT images in exactly the same position as the pCT ones.

## Conclusions

Adapt Anatomy has sufficient accuracy compared to other software solutions. In particular, there was no significant difference between CT and CBCT in terms of the DSC and OI readings for GTV; thus, we concluded that the Adapt Anatomy system achieved similar contouring accuracy on CBCT images as it did on CT ones. Therefore, we concluded that there is a possibility that Adapt Anatomy may be useful for performing ART on CBCT images. However, since its accuracy varies significantly for structures such as the esophagus, careful reviews by radiation oncologists are essential in clinical cases.

## References

[1] Dawson LA, Sharpe MB (2006). Image-guided radiotherapy: rationale, benefits, and limitations. Lancet Oncol.

[2] Hong TS, Tomé WA, Chappell RJ, Chinnaiyan P, Mehta MP, Harari PM (2005). The impact of daily setup variations on head-and-neck intensity-modulated radiation therapy. Int J Radiat Oncol Biol Phys.

[3] Li XA, Qi XS, Pitterle M (2007). Interfractional variations in patient setup and anatomic change assessed by daily computed tomography. Int J Radiat Oncol Biol Phys.

[4] Vestergaard A, Muren LP, Søndergaard J, Elstrøm UV, Høyer M, Petersen JB (2013). Adaptive plan selection vs. re-optimisation in radiotherapy for bladder cancer: a dose accumulation comparison. Radiother Oncol.

[5] Kataria T, Gupta D, Bisht SS (2014). Adaptive radiotherapy in lung cancer: dosimetric benefits and clinical outcome. Br J Radiol.

[6] Hatton JA, Greer PB, Tang C (2011). Does the planning dose-volume histogram represent treatment doses in image-guided prostate radiation therapy? Assessment with cone-beam computerised tomography scans. Radiother Oncol.

[7] Yang C, Liu F, Ahunbay E (2014). Combined online and offline adaptive radiation therapy: a dosimetric feasibility study. Pract Radiat Oncol.

[8] Song WY, Wong E, Bauman GS, Battista JJ, Van Dyk J (2007). Dosimetric evaluation of daily rigid and nonrigid geometric correction strategies during on-line image-guided radiation therapy (IGRT) of prostate cancer. Med Phys.

[9] Qin A, Sun Y, Liang J, Yan D (2015). Evaluation of online/offline image guidance/adaptation approaches for prostate cancer radiation therapy. Int J Radiat Oncol Biol Phys.

[10] Hansen EK, Bucci MK, Quivey JM, Weinberg V, Xia P (2006). Repeat CT imaging and replanning during the course of IMRT for head-and-neck cancer. Int J Radiat Oncol Biol Phys.

[11] Nuver TT, Hoogeman MS, Remeijer P, van Herk M, Lebesque JV (2007). An adaptive off-line procedure for radiotherapy of prostate cancer. Int J Radiat Oncol Biol Phys.

[12] Guckenberger M, Wilbert J, Richter A, Baier K, Flentje M (2011). Potential of adaptive radiotherapy to escalate the radiation dose in combined radiochemotherapy for locally advanced non-small cell lung cancer. Int J Radiat Oncol Biol Phys.

[13] La Macchia M, Fellin F, Amichetti M (2012). Systematic evaluation of three different commercial software solutions for automatic segmentation for adaptive therapy in head-and-neck, prostate and pleural cancer. Radiat Oncol.

[14] McDonald BA, Vedam S, Yang J (2021). Initial feasibility and clinical implementation of daily MR-guided adaptive head and neck cancer radiation therapy on a 1.5T MR-linac system: prospective R-IDEAL 2a/2b systematic clinical evaluation of technical innovation. Int J Radiat Oncol Biol Phys.

[15] Dice LR (1945). Measures of amount of ecologic association between species. Ecology.

[16] Tsuji SY, Hwang A, Weinberg V, Yom SS, Quivey JM, Xia P (2010). Dosimetric evaluation of automatic segmentation for adaptive IMRT for head-and-neck cancer. Int J Radiat Oncol Biol Phys.

[17] Brock KK, Mutic S, McNutt TR, Li H, Kessler ML (2017). Use of image registration and fusion algorithms and techniques in radiotherapy: report of the AAPM Radiation Therapy Committee Task Group No. 132. Med Phys.

[18] Fechter T, Adebahr S, Baltas D, Ben Ayed I, Desrosiers C, Dolz J (2017). Esophagus segmentation in CT via 3D fully convolutional neural network and random walk. Med Phys.

[19] Cohen RJ, Paskalev K, Litwin S, Price RA Jr, Feigenberg SJ, Konski AA (2010). Esophageal motion during radiotherapy: quantification and margin implications. Dis Esophagus.

[20] Palmer J, Yang J, Pan T, Court LE (2014). Motion of the esophagus due to cardiac motion. PLoS One.

[21] Voet PW, Dirkx ML, Teguh DN, Hoogeman MS, Levendag PC, Heijmen BJ (2011). Does atlas-based autosegmentation of neck levels require subsequent manual contour editing to avoid risk of severe target underdosage? A dosimetric analysis. Radiother Oncol.

[22] Chao KS, Bhide S, Chen H (2007). Reduce in variation and improve efficiency of target volume delineation by a computer-assisted system using a deformable image registration approach. Int J Radiat Oncol Biol Phys.

[23] Young AV, Wortham A, Wernick I, Evans A, Ennis RD (2011). Atlas-based segmentation improves consistency and decreases time required for contouring postoperative endometrial cancer nodal volumes. Int J Radiat Oncol Biol Phys.

[24] Hatton J, McCurdy B, Greer PB (2009). Cone beam computerized tomography: the effect of calibration of the Hounsfield unit number to electron density on dose calculation accuracy for adaptive radiation therapy. Phys Med Biol.

[25] Onozato Y, Kadoya N, Fujita Y (2014). Evaluation of on-board kV cone beam computed tomography-based dose calculation with deformable image registration using Hounsfield unit modifications. Int J Radiat Oncol Biol Phys.

[26] Giacometti V, King RB, Agnew CE, Irvine DM, Jain S, Hounsell AR, McGarry CK (2019). An evaluation of techniques for dose calculation on cone beam computed tomography. Br J Radiol.

